# Computational Aminoacyl-tRNA Synthetase Library Design for Photocaged Tyrosine

**DOI:** 10.3390/ijms20092343

**Published:** 2019-05-11

**Authors:** Tobias Baumann, Matthias Hauf, Florian Richter, Suki Albers, Andreas Möglich, Zoya Ignatova, Nediljko Budisa

**Affiliations:** 1 Institut für Chemie, Technische Universität Berlin, Müller-Breslau-Straße 10, 10623 Berlin, Germany; tobias.baumann@tu-berlin.de (T.B.); matthias.hauf@tu-berlin.de (M.H.); 2 Biophysikalische Chemie, Institut für Biologie, Humboldt-Universität zu Berlin, 10115 Berlin, Germany; flosopher@gmail.com (F.R.); andreas.moeglich@uni-bayreuth.de (A.M.); 3 Institute of Biochemistry and Molecular Biology, University of Hamburg, 20146 Hamburg, Germany; albers@chemie.uni-hamburg.de (S.A.); zoya.ignatova@chemie.uni-hamburg.de (Z.I.); 4 Lehrstuhl für Biochemie, Universität Bayreuth, 95447 Bayreuth, Germany; 5 Department of Chemistry, University of Manitoba, Winnipeg, MB R3T 2N2, Canada

**Keywords:** enzyme design, noncanonical amino acids, protein modification, directed evolution, mutagenesis, gene libraries, genetic code expansion, unnatural amino acids, protein engineering

## Abstract

Engineering aminoacyl-tRNA synthetases (aaRSs) provides access to the ribosomal incorporation of noncanonical amino acids via genetic code expansion. Conventional targeted mutagenesis libraries with 5–7 positions randomized cover only marginal fractions of the vast sequence space formed by up to 30 active site residues. This frequently results in selection of weakly active enzymes. To overcome this limitation, we use computational enzyme design to generate a focused library of aaRS variants. For aaRS enzyme redesign, photocaged *ortho*-nitrobenzyl tyrosine (ONBY) was chosen as substrate due to commercial availability and its diverse applications. Diversifying 17 first- and second-shell sites and performing conventional aaRS positive and negative selection resulted in a high-activity aaRS. This *Mj*TyrRS variant carries ten mutations and outperforms previously reported ONBY-specific aaRS variants isolated from traditional libraries. In response to a single in-frame amber stop codon, it mediates the in vivo incorporation of ONBY with an efficiency matching that of the wild type *Mj*TyrRS enzyme acylating cognate tyrosine. These results exemplify an improved general strategy for aaRS library design and engineering.

## 1. Introduction

### Computational Enzyme Redesign for Novel and Efficient aaRS Variants

**Figure A1:**
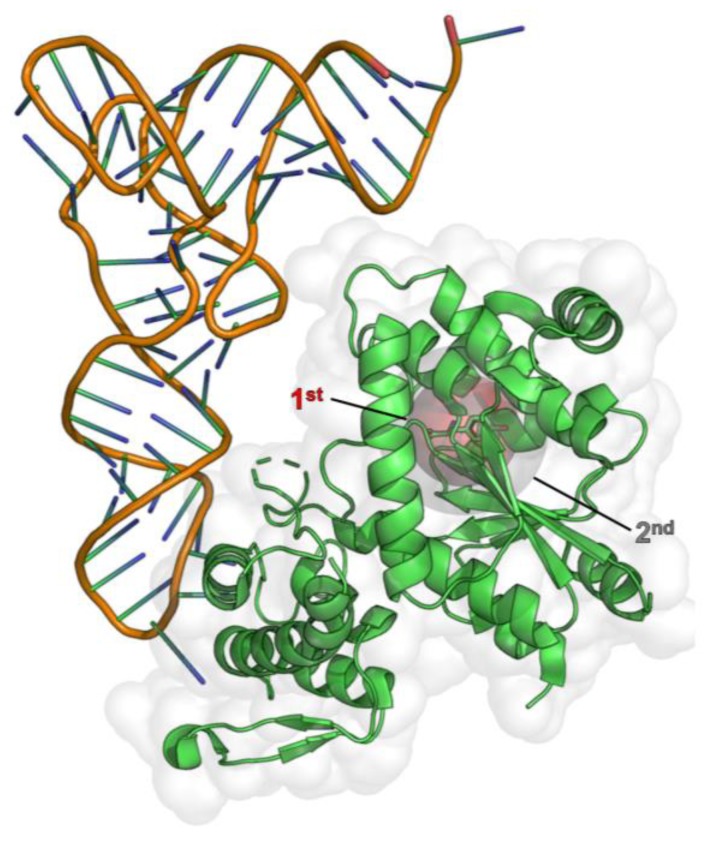
*Mj*TyrRS-tRNA^Tyr^ complex wild type structure with shells around the substrate Tyr (shown as sticks) highlighted. In the aaRS crystal structure with bound tRNA (PDB ID 1J1U), two shells—6 Å (red) or 9 Å (gray)—around the center of mass of the bound substrate are modeled. The solvent-accessible surface of the protein is indicated in light gray.

Aminoacyl-tRNA synthetases (aaRSs) are an important class of enzymes crucial for maintaining accuracy during translation of the genetic code [1]. The natural genetic code dictates which canonical amino acids are allowed for ribosomal translation. The expansion of their number beyond the canonical 20 requires a change or promiscuity in the substrate specificity of an aaRS, since these enzymes are crucial interpreters of the genetic code. To site-specifically introduce novel chemistries into proteins in both live cells and cell-free extracts, orthogonal pairs (o-pairs) consisting of an engineered aaRS and its cognate tRNA have been created in recent years. As part of a so-called orthogonal translation system (OTS), various aaRS/tRNA pairs allow installation of more than 200 noncanonical amino acids (ncAAs) into proteins, commonly in response to amber (i.e., TAG) stop codons [2,3,4]. To change the substrate specificity towards a desired ncAA, the active site of a given natural aaRS is redesigned, commonly via directed evolution employing a double-sieve positive/negative selection system [2]. For this purpose, aaRS gene libraries are traditionally generated by site-saturation mutagenesis (SSM), where, e.g., NNK codons (N: A/C/G/T; K: G/T) are used to create side chain diversity. A small number of residues in the first active site shell are rationally chosen based on their location within a few Angstroms from the natural substrate in the wild type enzyme (cf. Figure A1). For the majority of aaRS variants engineered thus far, *Methanocaldococcus jannaschii* tyrosyl-tRNA synthetase (*Mj*TyrRS) and *Methanosarcina mazei*/*barkeri* pyrrolysyl-tRNA synthetases (*Mm*PylRS or *Mb*PylRS, respectively) provided the template protein scaffold. It is their archaeal origin (and thus distant phylogeny) that has allowed their implementation in bacterial cells as o-pairs. Although many ncAA-specific aaRS variants have been generated this way, they often show low affinities towards the ncAA substrate and low overall aminoacylation activities for the two catalyzed steps of amino acid activation and cognate tRNA charging [4,5,6,7,8]. Evidently, the aminoacylation reaction requires a precise orientation of all involved substrates and is thus sensitive to even minimal perturbations [9]. At the same time, aaRS specificity must be sufficiently high to discriminate against canonical amino acids at intracellular concentrations. Low aaRS and thus o-pair efficiency is a major obstacle for cost-effective large-scale target protein production, not only creating the bottleneck for production, but also requiring the desired ncAA to be present in large excess. Consequently, it is desirable to generate highly efficient OTSs. Since gene library size is technically limited by the *E. coli* transformation efficiency to about 10^8^–10^9^ recombinants, only five to eight residues can be exhaustively tested at a time, even by sophisticated SSM approaches [10]. Early aaRS specificity engineering studies already indicated that first shell alteration by mutations may not be sufficient for the creation of efficient aaRS variants [11]. Selection of the active site residues targeted for mutagenesis can be facilitated by docking the new ncAA compound into the wild type active site using protein crystal structures. Commonly, selected positions are then randomized via insertion of NNK codons by various cloning strategies. Focusing solely on a few first-shell residues, however, would neglect the complex and subtle interplay of these with adjacent second-shell moieties [9]. Inspecting an aaRS active site usually identifies about 30 interdependent residues when counting first (<6 Å) and second (<9 Å) shells around the substrate (cf. Figure A1). To obtain a high-activity aaRS for a given ncAA, it is likely necessary to introduce compensatory mutations beyond residues directly contacting the novel substrate. Further, it is expected that mutations, which alter the aaRS active site architecture (for instance increasing its volume), impact the stability of the enzyme. In general, the considerable size of aaRSs with more than 300 residues, their multidomain composition and their commonly multimeric quaternary structure highlight the structural complexity of this enzyme type [9]. Assuming the aforementioned 30 residues are considered for enzyme redesign, a highly active sequence would need to be isolated from 20^30^ = 10^39^ total sequences. This is 30 orders of magnitude more than experimentally coverable by SSM libraries. As highlighted before [9], we hypothesize that the low activity often observed in engineered o-pairs can be attributed to this dramatic undersampling of relevant aaRS sequence space. Considering the number of contacts required to coordinate an amino acid substrate and to shape the aaRS active site appropriately, it may be challenging to obtain an aaRS suitable for efficient single- and especially multisite incorporation of *ortho*-nitrobenzyl tyrosine (ONBY) using this classical approach.

We attempt to overcome this dilemma by using computational enzyme design (CED) methodologies [12,13] in combination with established o-pair selection systems. Redesigning an aaRS is an example of the so-called ‘specificity redesign’ problem in CED [14]. Modern design protocols can rapidly scan through and evaluate a sequence space of size 10^39^ and above, but are not yet accurate enough to reliably and accurately identify a single defined sequence with high activity towards a desired enzyme substrate. However, they are capable of filtering large numbers of candidate sequences and of discarding the ones incompatible with substrate binding or the active site geometry. We thus propose a combined computational-experimental approach to aaRS engineering that goes beyond substrate docking via, e.g., AutoDock Vina [15] combined with SSM. In this approach, CED is first used to identify the most promising 10^8^–10^9^ aaRS sequences out of the total of ~10^39^ possibilities. Next, a focused gene library containing these computationally vetted sequences is constructed, and active variants are isolated using established aaRS selection strategies.

We deploy the Rosetta3 [12] enzyme design protocol [13] previously used for designing biocatalysts de novo [16,17,18,19] and for specificity redesign [20,21,22]. Thus, it can likely contribute to the successful design of novel o-pairs. ONBY was chosen as the target ncAA substrate. Upon irradiation with 365 nm light, the photolabile ONB moiety can be released—allowing the production of photoactivatable proteins [23,24,25]. Furthermore, this ncAA is commercially available, obviating the need for chemical multistep synthesis. So far, aaRSs for ONBY derived from *Mj*TyrRS and *Mb*PylRS were engineered using traditional methods [23,25]. Comparison with an aaRS for the same ncAA designed via our novel computational approach demonstrates the utility and benefit of CED methodology for efficient o-pair design.

## 2. Results

### 2.1. Computational Design of an aaRS Library

**Figure 1:**
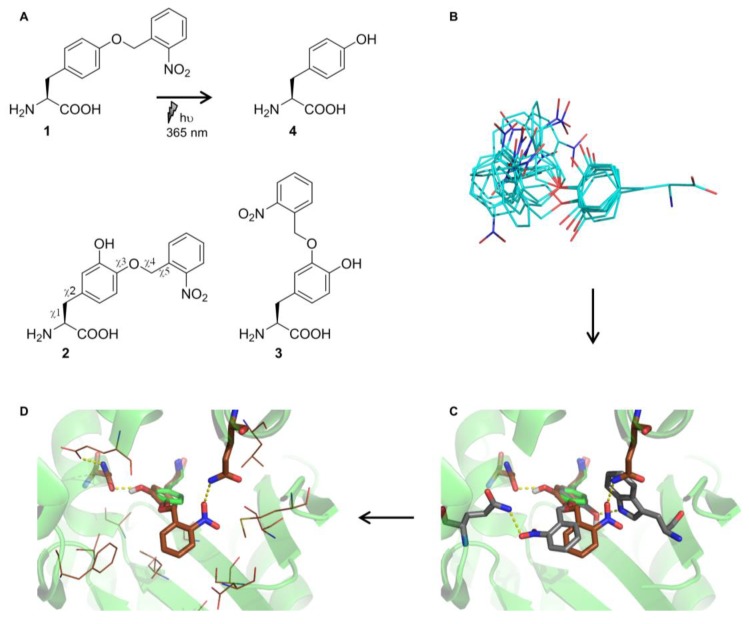
Compounds and computational design process to yield an aminoacyl-tRNA synthetase (aaRS) for genetic incorporation of *ortho*-nitrobenzyl tyrosine (ONBY). (**A**) ONBY (**1**), which can be photocleaved to yield Tyr (**4**), as well as chemical structures of *meta-* and *para-*ONB-DOPA (**2**, **3**). For **2**, rotation angles (χ1–5) are indicated. (**B**) Noncanonical amino acid (ncAA) ensemble of **2** diversified around χ3–5, with χ1–2 as observed in the *Mj*TyrRS wild type structure (PDB ID 1J1U) with the substrate tyrosine. (**C**) Matching stage of aaRS redesign: The ncAA substrate was placed in the 1J1U active site in orientations such that the core part overlaps with the substrate tyrosine (shown in green) and where specific interactions with the new polar groups can be formed. Two of the generated 143 matches are shown in gray and orange, respectively. (**D**) Design stage: Additional active site residues were redesigned to accommodate both the new ncAA substrate and the matched protein residues.

In previous work, we reported the computational redesign of the *Mj*TyrRS active site towards *ortho*-nitrobenzyl DOPA (ONB-DOPA), which is a photocaged analog of L-3,4-dihydroxyphenylalanine (DOPA) [26]. Two ncAA isomers had been focused on, where the photocleavable group is installed either at *meta* or *para* position (Figure 1A). Both had been used for functional selection, which yielded an efficient aaRS for *m*-ONB-DOPA. As ONBY differs from this ncAA substrate only at the *meta* position, a combined library compatible with both substrates was built. Throughout the design process, calculations were kept compatible with ONBY (see Materials and Methods). An ensemble of ncAA substrate models was created where the side-chain dihedral angles χ1 and χ2 (cf. Figure 1A) had values as the tyrosine (Tyr) substrate in the *Mj*TyrRS wild type (WT) crystal structure (PDB ID 1J1U) [27]; angles χ3–5 were diversified (Figure 1B). To then redesign the aaRS towards the ncAA, the Rosetta3 de novo enzyme design protocol (enzdes) [13] was used. In the matching stage of the computational protocol, placements of ONB-DOPA in 1J1U were first searched where the peptide backbone and core phenyl ring are superimposed onto the wild type tyrosine substrate, and the additional nitro (and *m-*hydroxy) groups are hydrogen-bonding with a (newly introduced) S/T/Y/N/Q/W residue (Figure 1C). In this way, 143 sequence-unique matches were found. Next, each match was redesigned 25 times (Figure 1D; note the specifics of the Rosetta molecular modeling approach [12]). During this design stage, mutations at 26 positions in and around the active site were considered. This corresponds to a theoretical sequence diversity of 20^26^ = 6.7 × 10^33^. From the resulting 3575 design models, 49 well-scoring ones (in terms of Rosetta energy) were selected. These bind the ncAA in a similar orientation with the ONB-ring interacting with polar residues at positions 167 and 180. To then generate the aaRS library, we compiled a sequence profile (i.e., a list of each observed identity at each designable position) from these 49 selected designs. This raw sequence profile (Supplementary Note 2) represented a sequence diversity of 8.4 × 10^17^, meaning that the computational design process so far reduced the sequence space in question by 16 orders of magnitude. However, these ~10^18^ sequences still far exceed the library complexity that can experimentally be handled [28]. Furthermore, as the design process had so far been carried out with ONB-DOPA, the library might not be optimally compatible with ONBY (Figure 1A). We thus curated the sequence profile (see Materials and Methods) and allowed wild type residues where contacts to the *m-*hydroxy group are predicted. With a final focused library (Table 1) size of ~10^8^, the computational process in combination with curation reduced the aaRS sequence space by 26 orders of magnitude.

### 2.2. Selection and Characterization of an aaRS Specific for ONBY

After synthesis and cloning, the aaRS gene library (Table 1) was subjected to alternating rounds of in vivo double-sieve selection [2] for ONBY. In order to evaluate and compare our computational approach, we used aaRS variants previously described for activation of this ncAA (see below). During two alternating selection rounds, the sequence converged to one aaRS variant bearing ten mutations, which we dubbed ONBYRS-1 (Table 1, DNA sequence in Supplementary Note 1). First, aaRS substrate specificity and discrimination against canonical amino acids were tested in vivo. Selective growth of clones expressing the ONBYRS-1/*Mj*tRNA_CUA_ o-pair and a chloramphenicol acetyltransferase (CAT) gene bearing two in-frame amber stop codons (construct CAT(2TAG)) was observed in presence of 1 mM ONBY on media containing ≥ 100 µg·mL^−1^ chloramphenicol (Cm) (Figure S1). Without ncAA supplementation, no TAG suppression was observed, as cells failed to grow in the presence of 20 µg/mL Cm. This strongly indicated that an efficient and selective aaRS for ONBY was selected.

**Figure 2:**
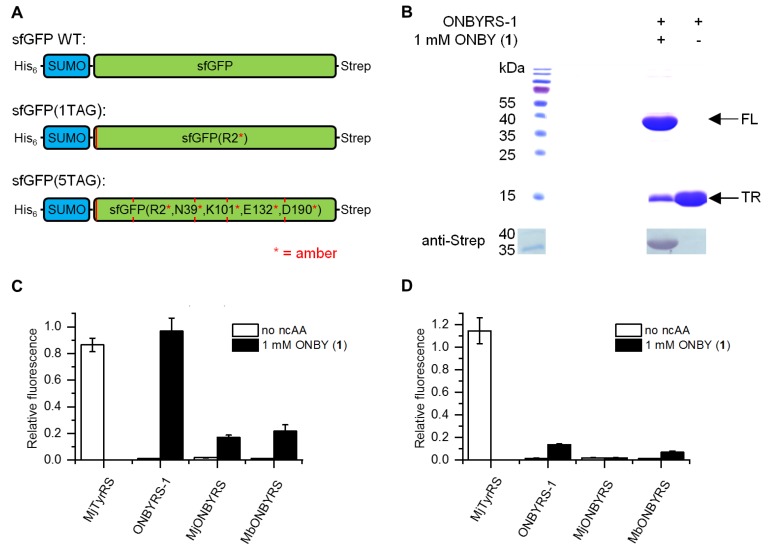
Characterization of ncAA incorporation by ONBYRS-1 into superfolder green fluorescent protein (sfGFP) reporter constructs and comparison with previously reported aaRSs (all expressed with corresponding tRNAs). (**A**) Schematic illustration of sfGFP reporter constructs for monitoring ncAA incorporation efficiency at one or five positions in sfGFP. Relative positions of the in-frame amber stop codons are indicated (red lines, the sfGFP WT control construct is free of these). (**B**) SDS-PAGE and anti-Strep Western blot analysis of sfGFP(1TAG) expression. Proteins were produced in *E. coli* BL21(DE3) using ONBYRS-1 in the presence or absence of 1 mM ONBY as indicated (FL = full-length, TR= truncated product). (**C**) Fluorescence reporter measurements for the incorporation of ONBY into sfGFP(1TAG) using *E. coli* BL21(DE3) cells. (**D**) Fluorescence reporter measurements for the incorporation of ONBY into of sfGFP(5TAG) using *E. coli* C321.ΔA.exp(DE3) cells. (**C**,**D**) Fluorescence values are normalized to strain-specific sfGFP WT fluorescence and to the OD_600_ of the bacterial culture. In case of ncAA supplementation, 1 mM ONBY was used. Data represent mean ± s.d. of biological triplicates.

In order to assess the fidelity and incorporation efficiency of the ncAA, reporter constructs were employed consisting of a small ubiquitin-like modifier (SUMO) sequence N-terminally fused with a superfolder green fluorescent protein (sfGFP) sequence. For detection or protein purification, an N-terminal His_6_-tag and a C-terminal Strep-tag II were further included. One or five in-frame amber stop mutations were introduced into sfGFP loop positions permissive for ncAA incorporation (cf. Figure 2A). In this way, the dynamic range of the reporter can be modulated, as five stop codons require a highly efficient OTS to produce detectable quantities of the full-length fluorescence protein. Combining the expression systems of a reporter (sfGFP(1TAG) or sfGFP(5TAG)) and the novel o-pair, constructs were expressed in two different host strains. First, *E. coli* BL21(DE3) served as a robust and routinely used recombinant expression host, where amber suppression competes with release factor 1 (RF1) translation termination activity. Second, the genomically recoded C321.ΔA.exp strain was employed as it lacks RF1, which should facilitate multisite amber stop codon suppression [29]. Two control constructs were employed as benchmark. First, wild type *Mj*TyrRS was used for tyrosine incorporation at amber sites, which is a highly efficient OTS. Second, the reporter construct without amber stop codons in the sfGFP sequence was built to test for maximum fluorescence signal and protein quantities obtainable in the absence of amber suppression (construct sfGFP WT). Reporter expression and amber suppression efficiencies were evaluated by two means, sfGFP intact cell fluorescence and purified protein yield.

With ONBYRS-1 and addition of ONBY, in-cell fluorescence of sfGFP(1TAG) was comparable to sfGFP WT as well as sfGFP(1TAG) with tyrosine incorporated via wild type *Mj*TyrRS/*Mj*tRNA_CUA_ (Figure 2C). In the setup for ONBY incorporation, suppression of five in-frame amber stop codons (reporter construct sfGFP(5TAG)) still reached fluorescence intensities ~15% of sfGFP WT (Figure 2D). Target proteins were purified by means of the N-terminal His-tag and yields were quantified. These correlate with the observed fluorescence intensity values and amount to 93 mg·L^−1^ sfGFP(1TAG) and 13 mg·L^−1^ sfGFP(5TAG) each containing ONBY (Table 1). Notably, these yields are markedly above those reported for the in vivo incorporation of ncAAs using most orthogonal pairs, including those based on *Mj*TyrRS [2,30]. SDS-PAGE and Western blot analyses confirmed the production of full-length target protein when expression of sfGFP(1TAG) takes place in presence of ONBY supplementation. Negligible traces of full-length protein observed in absence of ONBY demonstrate the selectivity of the aaRS (Figure 2C,D). Remarkably, only small amounts of truncated protein are visible in SDS-PAGE when ONBY is incorporated into sfGFP(1TAG). Despite the presence of RF1 in *E. coli* BL21(DE3), the evolved o-pair thus appears able to outcompete RF1-mediated translation termination in vivo. Mass spectrometry confirmed the incorporation of ONBY into sfGFP constructs at one or five positions (Figure S2). 

### 2.3. Comparison to Previously Reported aaRSs

**Figure 3:**
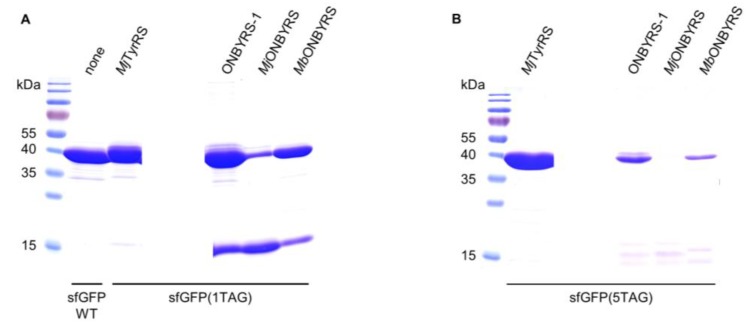
Coomassie-stained 15% SDS-PAGE of sfGFP(1TAG) and sfGFP(5TAG) constructs purified via Ni-NTA chromatography. (**A**) sfGFP WT and sfGFP(1TAG) constructs expressed in *E. coli* BL21(DE3) with different o-pairs (indicated above). (**B**) sfGFP(5TAG) constructs expressed in *E. coli* C321.ΔA.exp(DE3) cells with different o-pairs (indicated above). Equal volumes of pooled Ni-NTA elution fractions were used. The expected molecular weight of full-length sfGFP construct is ∼40 kDa (varies slightly with ncAA incorporation), while translation termination at position 2 of the reporter gene construct results in a truncation product ~12.5 kDa in size.

Thus far, two different aaRS variants for ONBY, based on either *Mj*TyrRS or *Mb*PylRS, respectively, have been reported (*Mj*ONBYRS and *Mb*ONBYRS) [23,25]. As both enzymes were evolved via conventional SSM, they can serve as a benchmark for our computational approach of ONBYRS-1 generation. We created the corresponding OTSs in a common genetic setup. As above, intact cell fluorescence and expression yields of the sfGFP amber suppression reporter constructs were determined. In comparison to ONBYRS-1, incorporation of ONBY into SUMO-sfGFP(1TAG) showed an approximately 6-fold or 4.5-fold decreased in-cell fluorescence when using *Mj*ONBYRS or *Mb*ONBYRS, respectively (Figure 2C). No significant fluorescence was observed for the incorporation of ONBY into sfGFP(5TAG) constructs using *Mj*ONBYRS, while fluorescence in the presence of *Mb*ONBYRS was about 2-fold lower than for ONBYRS-1 (Figure 2D). These data are consistent with the observed sfGFP expression yields (Figure 3, Table 2). To rule out that the observed higher stop codon suppression activity is caused by different expression levels of the aaRS variants in vivo, both *Mj*TyrRS-based enzymes, ONBYRS-1 and *Mj*ONBYRS, were expressed and purified. Since this resulted in similar aaRS yields of 18 mg and 12 mg per liter of culture, it indicates that the markedly higher ONBY incorporation and target protein production efficiencies using ONBYRS-1 result from superior enzymatic activity. In summary, the presented data demonstrate that the evolved ONBYRS-1 enzyme is substantially more efficient than the existing aaRSs and suggest that our computational approach to aaRS engineering can outperform classical SSM approaches.

**Figure 4:**
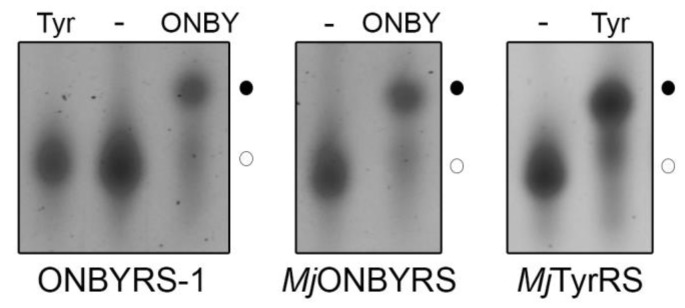
ONBY serves as a substrate of ONBYRS-1 and *Mj*ONBYRS. Representative in vitro aminoacylation of *Mj*tRNA_CUA_ by ONBYRS-1 (44 ± 8%, *n* = 6, left panel) and *Mj*ONBYRS (42 ± 9%, *n* = 10, middle panel) with ONBY, as well as by *Mj*TyrRS with Tyr (48 ± 12%, *n* = 15, right panel) analyzed by gel electrophoresis. In vitro transcribed, uncharged tRNA served as a control (-). ONBYRS-1 does not aminoacylate tRNA_CUA_ with tyrosine (Tyr, left panel). Aminoacyl-tRNAs (●) were detected by their slower migration compared to uncharged tRNA (○) in 6.5% acidic denaturing PAGE. tRNAs were visualized by SYBR gold staining.

To investigate the substrate specificity of the purified aaRS enzymes, in vitro aminoacylation of *Mj*tRNA_CUA_ by ONBYRS-1 and *Mj*ONBYRS was assessed. In vitro synthesized transcripts lack post-transcriptional modifications, that can stabilize tRNA structure [31]. However, structural studies with unmodified *E. coli* and yeast tRNA^Phe^ have shown that in vitro transcripts adopt an overall tertiary topology [32,33] and are aminoacylated to a similar extent as the native, fully modified tRNA [32,34]. Aminoacylated and non-aminoacylated tRNA fractions exhibit clear differences in their migration behavior on acidic gel electrophoresis, with aminoacyl-tRNA migrating slower (Figure 4). Aminoacylation levels are defined as the fraction of aminoacyl-tRNA from the total tRNA. As expected for an OTS, ONBYRS-1 was incapable of transferring L-Tyr to *Mj*tRNA_CUA_, suggesting that computational redesign combined with experimental aaRS selection yielded *Mj*TyrRS variants which lost their ability to activate and transfer the native substrate. Given that residues Y32 and D158, H bonding the phenolic oxygen of Tyr in the wild type enzyme, were both mutated, this was anticipated. Furthermore, the loss of Tyr specificity is a known feature of *Mj*TyrRS mutants evolved for new substrates [35] and a prerequisite for orthogonal translation. Under the chosen conditions, both *Mj*TyrRS-derived enzymes, ONBYRS-1 and *Mj*ONBYRS, showed an equal efficiency in transferring ONBY to *Mj*tRNA_CUA_ (~ 40%, see Figure 4), which resembles the efficiency of wild type *Mj*TyrRS in transferring its natural substrate Tyr to *Mj*tRNA_CUA_. These results likely show the state of plateau acylation in vitro. Amino acid activation and tRNA charging kinetics may differ between the aaRS variants, which may lead to the differences observed for the in vivo activity.

### 2.4. Active Site Modeling for ONBYRS-1 and Key Mutations

**Figure 5:**
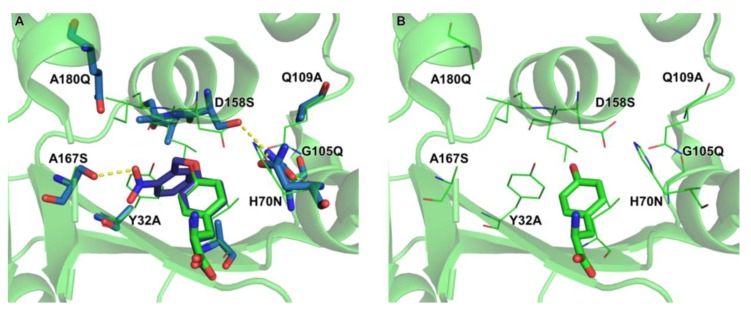
(**A**) ONBYRS-1 active site modeling with bound ONBY substrate. Mutations relative to wild type *Mj*TyrRS (PDB ID 1J1U, residues as thin lines) are indicated. The original orientation of the wild type substrate Tyr is shown in green, the similarly oriented ONBY is shown in purple. H bonds between S167 and the ONB nitro group as well as the H bond network between N70, Q105 and S158 are shown as dashed yellow lines. (**B**) Wild type *Mj*TyrRS structure for comparison, keeping ONBYRS-1 mutation sites indicated.

To get insight into the mode of substrate binding by ONBYRS-1, the aaRS active site with ten mutations was computationally modeled in complex with ONBY (Figure 5). The modeled structure suggests an H bond between the ncAA nitro group and the side-chain of S167, an aaRS mutation that was included in the library for this purpose. Another interesting feature is the H bond network between H70N, G105Q and D158S enabled by mutation Q109A. In wild type *Mj*TyrRS, D158 has H bonds with the substrate Tyr *p-*hydroxy group and Q109. As the *p*-hydroxy moiety is caged in ONBY, a first-shell mutation at position 158 is required, which in turn appears to necessitate compensatory mutations that preserve the structural integrity of the enzyme. 

**Figure 6:**
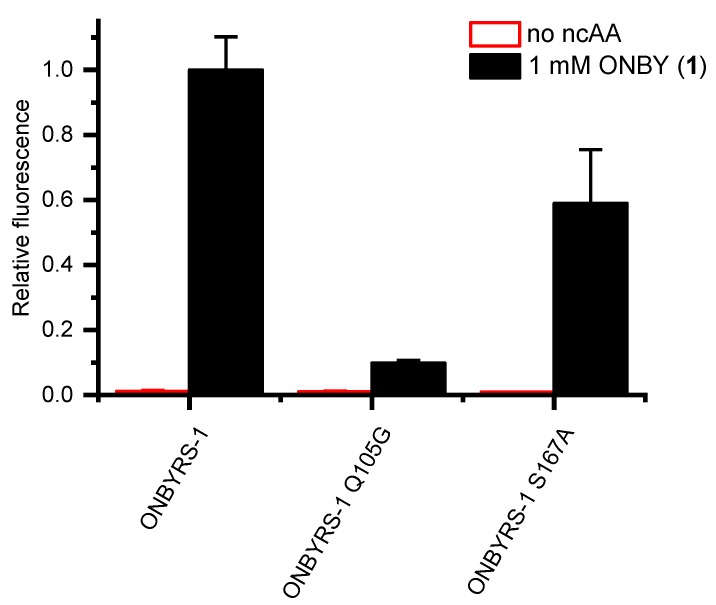
In-cell fluorescence measurements of construct sfGFP(1TAG) expressed in parallel to orthogonal translation systems with ONBYRS-1 and two aaRS mutants, respectively. Fluorescence values were normalized to the OD_600_ of the bacterial *E. coli* BL21(DE3) cultures. Data represent mean ± s.d. of biological triplicates.

This hypothesis was tested by reverting the two key mutations G105Q and A167S. The resulting ONBYRS-1 variants were then tested for in vivo stop codon suppression efficiency. Indeed, these two aaRS mutants revealed a drop in cellular sfGFP reporter fluorescence by ∼90% or 41%, respectively (Figure 6). This indicates that the interactions suggested by the model are indeed required for high ONBYRS-1 activity towards the ncAA substrate and for amber suppression efficiency attained in vivo.

### 2.5. Impact of aaRS Mutations on Thermal Stability

**Figure 7:**
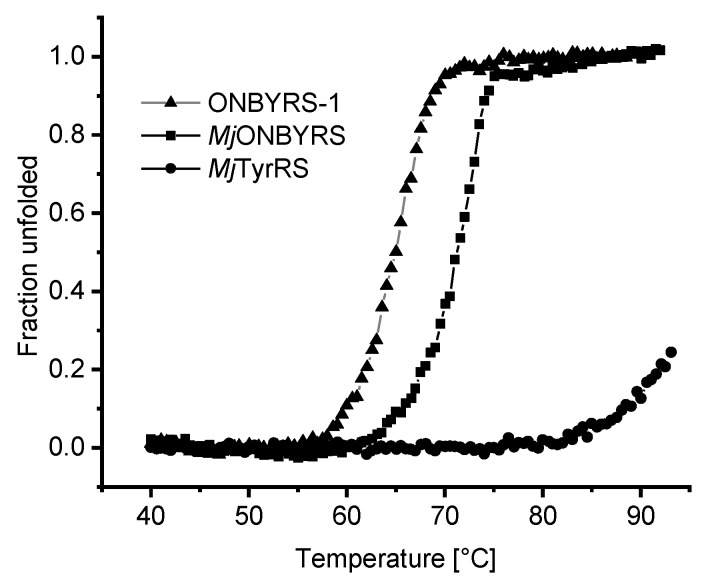
Stability of *Mj*TyrRS wild type and derived aaRS variants measured by circular dichroism (CD) spectroscopy. Thermal protein denaturation was performed in phosphate buffer while recording the CD signal at 222 nm. Due to the high thermostability of the wild type enzyme, the corresponding *T*_m_ value (>95 °C) cannot be accurately determined in the chosen setup. Calculated *T_m_* values for ONBYRS-1 and *Mj*ONBYRS are 64.8 °C and 71.0 °C, respectively.

Given that ONBYRS-1 contains ten mutations relative to the wild type enzyme, the impact of mutations on the thermal stability of the aaRS enzyme was investigated (Figure 7). As evident from thermal melting profiles, the stability of the protein scaffold is decreased by mutations altering the substrate specificity towards ONBY. Both *Mj*TyrRS-derived mutant proteins display a markedly decreased stability relative to the high melting point of the wild type enzyme. The latter is remarkably robust, as it could not be thermally unfolded to full extent within the used experimental setup.

## 3. Discussion

A noncanonical amino acid recruited by an expanded genetic code should be compatible with the following critical steps in the translation cycle; it should (i) participate in efficient tRNA aminoacylation while bypassing potential editing mechanisms, (ii) be capable of the formation of ternary complexes with elongation factor Tu and GTP (EF-Tu-GTP), and (iii) allow accommodation into the ribosome. Finally, (iv) it should readily participate in peptide bond formation at the peptidyl transfer center of the ribosome. This process of rewiring the cellular translation machinery also requires efficient cellular uptake of ncAAs, their intracellular metabolic stability and sustainable in-frame stop codon readthrough in context of the target mRNA sequence. In addition to ensuring o-pair orthogonality in the used host organism (i.e., the absence of cross-reactivity with components of the host translation machinery), the design of an ncAA-specific aaRS active site is the prime challenge for the site-specific incorporation approach [9]. In most cases reported thus far, the ncAA structure resembles that of the native substrate of the corresponding aaRS [2,36]. In line with this, engineered aaRS variants are often more promiscuous than the progenitor enzyme, which can be exploited to use a single enzyme for the incorporation of a variety of similar ncAA substrates. Generating enzymes for ncAAs with more different physicochemical properties thus should require extensive engineering efforts, involving several parts of the translation apparatus (see below).

The potential of computational tools for the identification of aaRS substrate compatibility has been realized early [37,38]. Docking ncAA substrates into aaRS active sites can help to choose residues for randomization [39,40], and computed ncAA-aaRS binding constants can be used to identify and rank substrate profiles [41,42]. In the present work, computational redesign of the *Mj*TyrRS active site towards recognition of a photocaged variant of tyrosine was performed. Several applications of ONBY have been reported, rendering this ncAA an attractive target [43,44,45,46,47,48,49,50]. A sequence space of ~10^34^ aaRS variants resulting from mutations at 26 sites was considered in silico. A focused library of 10^8^ members with sensible diversity at 17 positions was generated based on computational models. Through the combination of the computationally designed, focused aaRS library with established bacterial selection methods, a high-activity aaRS for ONBY was isolated. This *Mj*TyrRS-derived enzyme displays high substrate selectivity and efficiently discriminates against Tyr in vivo and in vitro. To our knowledge, ONBYRS-1 demonstrates the first multisite incorporation of photocaged tyrosine into recombinant proteins by means of an engineered o-pair. These results present important progress both in the field of aaRS/o-pair engineering and in the recombinant production of photocaged proteins for optochemical tools [51,52].

Comparing the activity data between ONBYRS-1 and the other two previously generated aaRSs for ONBY particularly demonstrates the benefits of our combined computational/screening approach over traditional aaRS engineering methods. One of the previous enzymes had been isolated from a six-position, the other from a five-position SSM library. Compared to these variants, our enzyme shows 6- and 4.5-fold improved stop codon suppression activity, despite the initial libraries used for in vivo selection having comparable sizes. Quite rarely observed, the engineered aaRS approaches wild type activity levels for suppression of a single in-frame amber stop codon. Moreover, it is worth noting that ONBYRS-1 features ten mutations. Even if all these ten positions had been known beforehand to be crucial for ONBY-specific activity, ONBYRS-1 could not have been obtained using traditional methods, as a ten-residue SSM library (size > 10^13^) would be undersampled by several orders of magnitude during the in vivo selection stage. We believe the key advantage of our combined approach to be that for every aaRS active site position, the computational stage suggests only a limited set of appropriate mutations. Thus, a library containing sensible focused diversity at each of these positions can be devised for the target ncAA substrate. More aaRS residues can be considered (and thus targeted by mutagenesis) than if every single position is completely randomized.

Notably, it is quite likely that implementing second-shell mutations in the computational process increased chances for the isolation of a functional aaRS. The need to accommodate mutations of first-shell residues essentially extends the design shell beyond the immediate ligand shape. The broader sequence space sampled by the combined approach could also allow generation of aaRS variants for substrates inaccessible for libraries only encompassing residues in direct contact with the natural substrate. Molecular modeling was possible using structures of the wild type enzyme and the aaRS variant specific for *m-*ONB-DOPA selected from the same library. In particular, the library contained large-to-small second-shell mutations (e.g., Q109A). In general and fitting to the requirements of a larger substrate, several mutations to smaller residues (Y32A, L65A, I159A) appear crucial to create space within the active site for larger substituents at the *para* position of the substrate. According to our modeling, the substitution A167S allows coordination of the ONB nitro group. The importance of this mutation is further evident from the activity loss upon reversal. As another means of identifying beneficial mutations distant to the immediate substrate binding pocket, aaRS genes have been subjected to error-prone PCR mutagenesis and DNA shuffling [8,53,54]. This could in principle also lead to the discovery of compensatory and second-shell mutations. In addition to accommodation and alignment of the ncAA substrate, these can also enhance aaRS performance by improvements of protein folding, stability and solubility. The wild type *Mj*TyrRS scaffold, as typical for a protein derived from a hyperthermophilic host, is remarkably resistant towards thermal unfolding. This likely provides an excellent starting point for evolution towards new function [55]. Relative to this starting enzyme, *Mj*ONBYRS bearing five and ONBYRS-1 bearing ten mutations show a decreased thermal stability. Evidently, engineering of the aaRS scaffold towards ONBY activation decreased thermal stability. Given the requirement to bind and activate a larger substrate, this effect of the corresponding mutations is not unexpected. Based on the in vivo performance of ONBYRS-1 at 37 °C, it seems unlikely that specificity reengineering shifted the protein stability to critically low values. Still, it cannot be ruled out that other aaRS engineering efforts reach this limit, impeding selection of a highly functional aaRS or necessitating compensatory mutations.

We note that in this work, the existing Rosetta3 computational protocols were essentially used out-of-the-box, requiring little to no modification towards the peculiarities of aaRSs or the ncAA at hand. aaRS engineering endeavors commonly employ established o-pairs as a starting point, for which crystal structures are readily available (e.g., *Mj*TyrRS and *Mm/Mb*PylRS) [27,56]. We thus believe that our combined structure-based computational and in vivo selection approach could be easily deployed in future aaRS redesign projects, and broadly aid the development of high-activity aaRS variants for desirable ncAAs. Such effective enzymes will become even more important as recent advancements, like RF1 removal and genome-wide amber codon emancipation [29,57], have enabled multisite incorporation of ncAAs into proteins in *E. coli*. Whereas ncAA incorporation has traditionally focused on single site modifications, multisite incorporation will allow the biosynthesis of protein-based biomaterials, such as mussel-inspired adhesives [26], or the design of proteins bearing novel chemical and physiochemical properties now conferred by multiple ncAAs. In all these scenarios the availability of high-activity aaRS enzymes is indispensable. Certainly, extending ncAA incorporation in this direction requires well-expressing protein scaffolds with multiple permissive positions as for example described for elastin-like polypeptides (ELP) [58]. Starting from such scaffolds holds great promise for the production of larger quantities of site-specifically photocaged proteins. Concerning the range of ncAA-modified target proteins, it should be noted that procedures to build backbone-dependent rotamer libraries for ncAAs and their usage in Rosetta have been published, which facilitate molecular protein modeling and (re)design [59].

Together with *Mj*TyrRS, the likewise archaeal PylRS pairs (*Mm/Mb*PylRS) fuel the development of most ncAA incorporation systems. Both aaRS scaffolds hold advantages and disadvantages. Whereas variants of *Mj*TyrRS frequently retain high activity and thus allow for efficient production of ncAA-modified proteins, these enzymes cannot be used in mammalian cells due the lack of orthogonality. PylRS variants fill this gap, allowing application of derived o-pairs in various organisms, including key model organisms such as transgenic mice, zebrafish, and fruit flies [60]. However, originating from an enzyme naturally evolved to incorporate pyrrolysine (Pyl) at only few positions in the proteome [61], PylRS-derived aaRS enzymes commonly display low enzymatic efficiency, limiting the obtainable target protein yields and the number of in-frame stop codons which can be suppressed. With efforts to alleviate this limitation underway [62,63], computational redesign of this aaRS scaffold towards new substrates is also very promising. In a system engineering approach to optimize ncAA incorporation, the anticodon interaction domain of *Mj*TyrRS and the EF-Tu amino acid binding pocket have been targeted [64]. Again, the number of involved residues was too large for the classical SSM approach, tackled by the creation of distinct libraries and separated evolution experiments. Including the role of the orthogonal tRNA in the modeling process, these areas of OTS engineering could also benefit from computational approaches. Ultimately, a deep structural and mechanistic understanding of amino acid substrate recognition and discrimination by natural and engineered aaRSs may help to understand the origin of the genetic code [7,65].

## 4. Materials and Methods

### 4.1. Computational Design Procedures

Our previous work for the computational design of an aaRS for *m-*ONB-DOPA illustrates the basic procedure [26]. To build an initial model of *p*-ONB-DOPA (adjustments to ONBY below), tyrosine coordinates taken from the tyrosine substrate of the 1J1U crystal structure were loaded into Avogadro [66], and the extra atoms added. This model was then diversified around the χ3–5 angles using OpenEye Omega [67], yielding 500 conformers. In addition, for every conformer, a version where χ2 was rotated by 180° was created to allow for the *m*-hydroxy group to be on either side of the *p*-ONB substituent, thus yielding a conformer ensemble of 1000 members (cf. Figure 1B).

The Rosetta3 enzyme design protocol [13] consists of two main stages: first, 2–4 residues making critical interactions with the novel substrate are placed in the active site according to geometric criteria (so-called ‘matching’), and afterwards, the remaining first- and second-shell residues are redesigned using a Monte Carlo rotamer placement algorithm in combination with an atomic resolution energy function. A matching run with three match constraints was set-up: (1) between a glutamine and the ONB-DOPA carboxylate, (2) between S/T/Y/N/Q/W and the ONB-DOPA nitro group, and (3) between S/T/Y/N/Q/W and the *m*-hydroxy group. Parameters for constraint 1 were chosen according to the orientation between Q173 and the substrate-tyrosine in 1J1U, and parameters for constraints 2 and 3 were chosen to form standard H bonds. Matching was then carried out using a minimized version of 1J1U as scaffold, with Q173 in its native rotameric state being the only residue sampled for match constraint 1. For constraints 2 and 3, all residue identities at all scaffold positions were allowed. As χ1–2 were not varied in the ONB-DOPA ensemble, and only Q173 was allowed for constraint 1, all 143 matches featured the core part of ONB-DOPA superimposed onto the 1J1U substrate tyrosine, with the *p*-ONB group exploring the active site pocket. Command lines and input files are in the Supplementary Information.

For the design stage, the Rosetta3 enzyme design workflow as previously described [13] was implemented in RosettaScripts [68]. To ensure integrity of catalytic residues, in addition to the match constraints, interactions between the ONB-DOPA peptide backbone and Y151/Q155 were also constrained to their 1J1U-observed values. Residues within 9 Å of any ligand atom were considered designable, with a resfile specifying several (e.g., catalytic) residues to be kept constant. Filtering of resulting designs was according to ligand score (to ensure binding), constraint score (to ensure preservation of catalytic geometry), and RMS of active site residues after repacking without ligand (to ensure preorganization). Of the 3575 design models, 83 passed all filters. These designs were then clustered according to substrate orientation, with four distinct orientations observed. We decided to construct the aaRS library based on the largest cluster (49 designs), as Rosetta had converged on it most frequently. A sequence profile of these 49 designs yielded a diversity of 8.4 × 10^17^ (Supplementary Note 2). To reduce library size to an experimentally manageable ~ 10^8^, all residues appearing at low frequencies (< 5%) were removed. Conservative mutations at distal regions of the active site (e.g., L66I) were also removed. If similar mutations were present at a particular position (e.g., S/T or V/I/L), only one of these was considered. To ensure compatibility with ONBY, wild type residues were also contained in the library at positions where mutations to contact the *m*-hydroxy group were introduced.

The design stage command line and all input files are in the Supplementary Information. The sequence profile of the 49 designs selected for library generation was created using a custom python script [69]. 

### 4.2. Noncanonical Amino Acid

ONBY hydrochloride was purchased from Activate Scientific (Prien, Germany). 

### 4.3. Library Construction and Selection

Detailed plasmid construction was described previously [26]. Briefly, the aaRS gene library including 17 mutagenic positions was synthesized (GeneArt, Thermo Fisher Scientific, Regensburg, Germany). Compared to *Methanocaldococcus jannaschii* wild type TyrRS, all aaRS variants carry a fixed D286R mutation [27]. The gene library was amplified via PCR, digested with NcoI and PstI and cloned into plasmid pBU18-GFP replacing the GFP variant gene under control of the *E. coli* glnS’ promoter and terminator in order to prevent the presence of wild type *Mj*TyrRS sequences in case of incomplete digestion. Transformation of electrocompetent *E. coli* DH10B cells yielded 4 × 10^9^ colonies, thus covering the theoretical diversity of the library of 1.6 × 10^8^ by > 99% [10]. Sanger sequencing of 12 randomly chosen clones verified the quality of the library. After library DNA isolation, two alternating rounds of positive and negative selections were conducted as described earlier [2]. Briefly, *E. coli* DH10B cells containing the positive selection plasmid (pPAB26 carrying an optimized mutant *Mj*tRNA_CUA_ (amber suppressor tRNA with CUA anticodon derived from Methanocaldococcus jannaschii tyrosyl-tRNA) [70] under control of the proK promoter and terminator as well as a chloramphenicol resistance (CmR) gene harboring amber stop codons at positions Q98 and D181) were transformed with the plasmid library. Cells were plated on New Minimal Medium (NMM) [71] agar plates (24 cm × 24 cm) supplemented with 1 mM of ONBY and 30, 50 or 70 µg·mL^−1^ Cm. Plates were incubated at 37 °C for 48 h in the dark. Plasmid library DNA was purified from collected cells. For negative selection, cells containing the negative selection plasmid (pBU26, encoding the barnase gene bearing amber stop codons at positions Q2 and D44 placed under control of an arabinose promoter and rrnC terminator as well as the *Mj*tRNA cassette) were transformed with plasmid library DNA and plated on LB agar plates containing ampicillin, kanamycin, and 0.002% (*w*/*v*) L-arabinose. After incubation at 37 °C for 16 h in the dark, cells were collected. The plasmid library DNA was purified and used for the next selection round. After the second selection round, 30 clones specifically growing in presence of ONBY were randomly selected. Cells were tested for growth on NMM agar plates containing 20 or 100 µg·mL^−1^ cm, either in presence or absence of 1 mM ONBY. Eight clones were selected which survived at the highest Cm concentration in the presence of ONBY, but which did not survive or marginally grew at 20 µg·mL^−1^ Cm in the absence of the amino acid. Plasmids encoding the *Mj*TyrRS variants were isolated and the aaRS genes sequenced. All potential aaRS hits had identical sequences which was termed ONBYRS-1. The synthetase gene was subcloned into pULTRA or pBU16 vectors to generate pULTRA-ONBYRS-1 and pBU16-ONBYRS-1, respectively.

### 4.4. Bacterial Strain Construction

C321.ΔA.exp [29] was a gift from George Church (Addgene plasmid # 49018). To enable inducible protein production by the T7 promoter system, the lambda DE3 lysogen was chromosomally introduced via the λDE3 Lysogenization Kit (Novagen, Merck KGaA, Darmstadt, Germany). Introduction was verified via PCR. 

### 4.5. Analysis of sfGFP Expression by Intact Cell Fluorescence

Intact cell fluorescence measurements of sfGFP reporter constructs were performed on an Infinite M200 microtiter plate reader (Tecan, Männedorf, Switzerland). *E. coli* strain BL21(DE3) was employed for measurements of the reporter construct with one amber codon and C321.ΔA.exp(DE3) [29] for measurements with five amber codons, respectively. Briefly, bacterial strains transformed with an OTS on pBU16 plasmids and the corresponding pET-28 sfGFP reporter plasmid were inoculated from glycerol stocks and grown to saturation overnight in liquid cultures. Each microtiter plate well contained cultures at 1:100 dilution in ZYP-5052 autoinduction medium [72] supplemented with 100 µg·mL^−1^ kanamycin, 100 µg·mL^−1^ spectinomycin and 1 mM ONBY (in case of ncAA supplementation) in a final volume of 300 µL. The 96-well µ-plates (Ibidi, Martinsried, Germany) were covered with a gas-permeable foil (Sigma-Aldrich, St. Louis, MO, USA) and incubated with orbital shaking for 17 h at 37 °C. OD_600_ and fluorescence intensities were directly measured, the latter via bottom reading using excitation and emission wavelengths of 481 nm and 511 nm, respectively, and a fixed manual gain of 85. Fluorescence intensity values were normalized to the corresponding OD_600_. Biological triplicates were used for each measurement. 

### 4.6. Protein Expression and Purification

sfGFP(1TAG) and sfGFP(5TAG) constructs were expressed in BL21(DE3) or C321.ΔA.exp(DE3) cells. Target proteins were purified via Ni-NTA chromatography. Relevant elution fractions were pooled and protein concentration was calculated by measuring the absorbance at 488 nm of purified proteins [73]. aaRS variants were expressed and purified via Ni-NTA and hydrophobic interaction chromatography as described [26].

### 4.7. Western Blot Analyses

sfGFP protein constructs were separated on 12% or 15% SDS polyacrylamide gels. After transfer onto nitrocellulose membranes, proteins carrying a His_6_-tag were detected using rabbit anti hexahistidine antibody (Abcam, Cambridge, UK) and goat anti-rabbit IgG alkaline phosphatase-conjugated antibody (Abcam, Cambridge, UK). Proteins carrying a Strep-tag II were detected via mouse anti Strep-tag II antibody (Merck Millipore, Darmstadt, Germany) and goat anti-mouse IgG (whole molecule) alkaline phosphatase-conjugated antibody (Sigma-Aldrich, St. Louis, MO, USA). Blots were developed using nitro-blue tetrazolium (NBT) and 5-bromo-4-chloro-3’-indolyphosphate (BCIP) staining (both reagents from Carl Roth, Karlsruhe, Germany). 

### 4.8. Mass Spectrometry

Intact proteins were analyzed by liquid chromatography-electrospray ionization mass spectrometry (LC-ESI-MS) using a QTOF 6530 instrument (Agilent, Waldbronn, Germany). Spectra deconvolution was performed with Agilent MassHunter Qualitative Analysis software employing the maximum entropy deconvolution algorithm.

### 4.9. Thermal Protein Unfolding

Stability of aaRS proteins against thermal unfolding was determined using circular dichroism spectroscopy. Protein solutions (20 µM) in 20 mM sodium phosphate buffer containing 150 mM KCl were subjected to a temperature ramp of 1 °C min^−1^ while monitoring the CD signal at 222 nm. Quartz cuvettes (Hellma, Müllheim, Germany) with a path length of 1 mm and a J-815 spectrometer (Jasco, Pfungstadt, Germany) equipped with a Peltier element were used. *T_m_* values were obtained as described [74] with curve-fitting errors below ±0.08 °C.

### 4.10. In Vitro Transcription and Aminoacylation of tRNA

As for in vivo experiments, an optimized mutant of *Mj*tRNA_CUA_ [70] was used throughout. The template for T7-promoter driven transcription of *Mj*tRNA_CUA_ was generated by annealing and primer extension of two overlapping DNA oligonucleotides covering the tRNA sequence including an upstream T7 promoter with the following sequences; forward, 5’-GGATCCTAATACGACTCACTATACCGGCGGTAGTTCAGCAGGGCAGAACGGCGGACTC-3’, reverse, 5′- TGGTCCGGCGGAGGGGATTTGAACCCCTGCCATGCGGATTTAGAGTCCGCCGTTCTGC-3’. Equimolar concentrations of primers (24 µM each) were denatured for 2 min at 95 °C and incubated for 3 min at room temperature in 20 mM Tris-HCl (pH 7.5). Primer extension was performed in 250 mM Tris-HCl (pH 8.3, containing 250 mM KCl, 20 mM MgCl_2_, 50 mM DTT, 0.4 mM dNTPs) and 4 U/μL RevertAid Reverse Transcriptase (Thermo Fisher Scientific, Waltham, MA, USA ) for 40 min at 37 °C. The dsDNA template was extracted using phenol/chloroform (Carl Roth, Karlsruhe, Germany), precipitated with ethanol and resuspended in DEPC-H_2_O. The in vitro T7-driven transcription was performed with 1 μg template DNA in 40 mM Tris-HCl (pH 7.0, containing 6 mM MgCl_2_, 10 mM DTT, 10 mM NaCl, 2 mM spermidine, 2 mM NTPs, 1.25-5 mM GMP), and 30 U T7 RNA polymerase (Thermo Fisher Scientific, Waltham, MA, USA) overnight at 37 °C. tRNA was precipitated with ethanol and purified using 10% preparative denaturing PAGE. Pure tRNA was eluted in 50 mM KOAc, 200 mM KCl (pH 7.0) by constant shaking at 1000 rpm overnight, precipitated with ethanol and resuspended in DEPC-H_2_O.

tRNA folding and in vitro aminoacylation reactions were performed as described previously [75] with 1 μM of the corresponding purified aaRS and 1 mM of amino acid added. Aminoacylated tRNAs were precipitated with ethanol and directly dissolved in acidic RNA loading dye (0.1 M NaOAc pH 4.8, containing 8 M urea, 5% glycerol, 0.025% (*w*/*v*) bromophenol blue, and 0.025% (*w*/*v*) xylene cyanol FF). Charged and uncharged tRNA fractions were separated on denaturing acidic 6.5% PAGE (8 M urea, 0.1 M NaOAc, pH 5) at 4 °C. *Mj*tRNA_CUA_ was visualized by SYBR gold (Invitrogen, Carlsbad, CA, USA) staining.

## Figures and Tables

**Table 1:** aaRS library resulting from curated Rosetta calculations and sequence of the experimentally selected *Mj*TyrRS variant.

*Mj*TyrRS Position	Library	ONBYRS-1
Y32	A, S	**A**
L65	A, I, L, F, S	**A**
A67	A, Q	A
L69	L, K, G, W	L
H70	A, N, S	**N**
G105	G, A, Q	**Q**
F108	F, L	F
Q109	A, Q, Y	**A**
M154	E, M, T, G	M
D158	A, G, S	**S**
I159	A, G, S, I	**A**
L162	A, M	**A**
V164	A, T, V	V
A167	N, Q, G, S	**S**
H177	A, H, Q, Y	H
A180	A, Q	**Q**
V188	N, Q, T, V	V

Wild type aaRS residues depicted in black. Mutations are classified according to the following color code: first-shell mutations: space/packing around substrate (red), polar interactions with ONB nitro group (blue); second-shell mutations: space/packing around first-shell mutations (orange), polar interactions with first-shell mutations (light blue). Additional mutations beyond these classifications shown in gray. All *Mj*TyrRS variants used in this study bear a fixed D286R mutation to improve amber suppression efficiency [27].

**Table 2:** Purified protein yields of sfGFP constructs expressed in different bacterial strains.

Protein Construct	*E. coli* Strain	O-Pair	ONBY	Yield (mg·L^−1^)
**sfGFP WT**	BL21(DE3)	-	-	89 ± 16
**sfGFP(1TAG)**	BL21(DE3)	*Mj*TyrRS	-	81 ± 23
		ONBYRS-1	+	93 ± 9
		*Mj*ONBYRS	+	7 ± 2
		*Mb*ONBYRS	+	21 ± 2
**sfGFP(5TAG)**	C321.ΔA.exp(DE3)	*Mj*TyrRS	-	89 ± 12
		ONBYRS-1	+	13 ± 2
		*Mj*ONBYRS	+	n.d.
		*Mb*ONBYRS	+	5 ± 1

Protein yields per liter of bacterial culture were determined by measuring absorbance at 488 nm which originates from sfGFP chromophore absorption. Samples were purified via Ni-NTA affinity chromatography. n.d.: not determined since yields were too low. Data represent mean ± s.d. of biological triplicates.

## Supplementary Materials

Supplementary materials can be found at https://www.mdpi.com/1422-0067/20/9/2343/s1.

## Abbreviations

aaRS	aminoacyl-tRNA synthetase
CAT	chloramphenicol acetyltransferase
CD	circular dichroism
CED	computational enzyme design
Cm	chloramphenicol
DOPA	L-3,4-dihydroxyphenylalanine
ELP	elastin-like polypeptide
*Mb*	*Methanosarcina barkeri*
*Mj*	*Methanocaldococcus jannaschii*
*Mm*	*Methanosarcina mazei*
ncAA	noncanonical amino acids
ONB-DOPA	*ortho*-nitrobenzyl L-3,4-dihydroxyphenylalanine
ONBY	*ortho*-nitrobenzyl L-tyrosine
o-pair	orthogonal pair
OTS	orthogonal translation system
PylRS	pyrrolysyl-tRNA synthetase
RF1	release factor 1
sfGFP	superfolder green fluorescent protein
SSM	site-saturation mutagenesis
SUMO	small ubiquitin-like modifier
Tyr	L-tyrosine
TyrRS	tyrosyl-tRNA synthetase
WT	wild type
