# Supplementary material for: Computational Aminoacyl-tRNA Synthetase Library Design for Photocaged Tyrosine
Source: Int J Mol Sci. 2019 May 11;20(9):2343. doi: 10.3390/ijms20092343 (PMC6539999; doi:10.3390/ijms20092343)
Supplement: Supplementary file 1 [file ijms-20-02343-s001.pdf]

Computational aminoacyl-tRNA synthetase library design for photocaged tyrosine

Supplementary Information

Tobias Baumann[1,†], Matthias Hauf[1,†], Florian Richter[2,3], Suki Albers[4], Andreas Möglich[2,5], Zoya Ignatova[4] and Nediljko Budisa[1,6,*]

1 Institut für Chemie, Technische Universität Berlin, Müller-Breslau-Straße 10, 10623 Berlin (Germany)
2 Biophysikalische Chemie, Institut für Biologie, Humboldt-Universität zu Berlin, 10115 Berlin (Germany)
3 present address: Bayer AG, Pharmaceuticals, Protein Engineering and Assays, Nattermannallee 1, 50829 Köln (Germany)
4 Institute of Biochemistry and Molecular Biology, University of Hamburg, Hamburg (Germany)
5 Lehrstuhl für Biochemie, Universität Bayreuth, Bayreuth (Germany)
6 Department of Chemistry, University of Manitoba, R3T 2N2 Winnipeg, MB (Canada)
* Correspondence: nediljko.budisa@tu-berlin.de
† These authors contributed equally to this study.

Supplementary Results

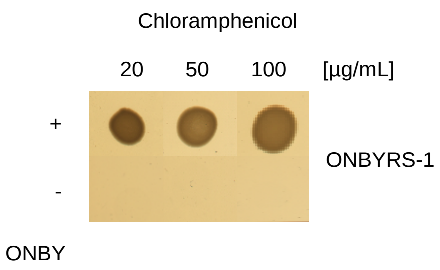

Supplementary Figure 1. Growth assay of *E. coli* DH10b cells expressing CAT(2TAG) and the ONBYRS-1 o-pair. As a functional OTS, ONBYRS-1 and *Mj*tRNA$^{Tyr}_{CUA}$ were expressed in presence (+) and absence (-) of 1 mM ONBY at different Cm concentrations. Bacterial growth was observed up to 100 µg mL^{-1} Cm in the presence of ONBY, while no relevant growth was observed in the absence of ONBY. Cells were grown in LB medium, diluted to an OD_{600} of 1.0, followed by spotting of 2 µL onto NMM agar supplemented with Amp, Kan (for maintenance of the OTS and selection plasmids) and different concentrations of Cm.

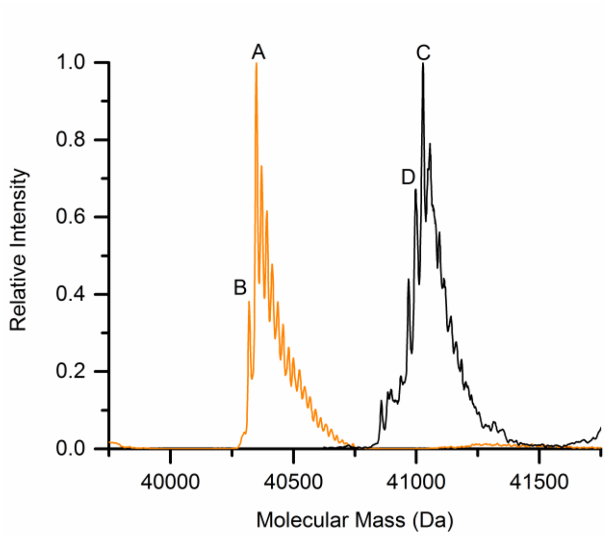

Supplementary Figure 2. Deconvoluted ESI-MS spectra of sfGFP constructs with incorporation of ONBY. ESI-MS of ONBY incorporated into SUMO-sfGFP(1TAG) and SUMO-sfGFP(5TAG) using ONBYRS-1. The found and expected masses are as follows: Yellow trace: SUMO-sfGFP(1ONBY), (A) observed: 40350.4 Da, expected: 40350.6 Da. Peak (B) is consistent with reduction of the nitro group to an amine as reported previously[1,2]. Black trace: SUMO-sfGFP(5ONBY), (C) observed: 41056.6 Da, expected: 41057.3 Da. Peak (D) and adjacent peaks at lower masses are consistent with reduction of the nitro group to an amine.

Supplementary Note 1. DNA sequences

Sequences of SUMO-sfGFP amber suppression reporter constructs on pET-28 plasmid vectors as well as details for the OTS expression and o-pair selection constructs were reported previously.[3]

Sequence of ONBYRS-1 evolved in this study. Mutations in comparison to *Mj*TyrRS(D286R) are highlighted in yellow.

atggatgaatttgaaatgattaaacgcaacaccagcgaaatcattagcgaagaagaactgcgcgaagtcctgaaaaaagatgaaaaaagcgccgcaattggctttgaaccg
agcggtaaaattcatctgggtcattatctgcaaatcaaaaaaatgattgatctgcaaaacgcaggctttgatattatcattgcactggccgatctaaacgcctatctgaatcagaaa
ggcgaactggatgaaattcgcaaaattggcgactataacaaaaaagtgtttgaggcaatgggcctgaaagccaaatatgtgtatcaaagcgaatttgccctggataaagattat
accctgaacgtgtatcgtctggcactgaaaaccaccctgaaacgtgcacgtcgtagcatggaactgattgcacgtgaagatgaaaatccgaaagttgccgaagtgatctatccg
attatgcaggtgaacagcgcacattatgccggcgtcgatgttagcgtgggtggtatggaacagcgcaaaatccatatgctgcaacgtgaactgctgccgaaaaaagtcgtgtgc
attcataatccggttctgaccggtctggatggtgaaggcaaaatgagcagcagcaaaggtaactttattgccgttgatgatagtccggaagaaattcgtgccaaaatcaaaaaag
catattgtccggcaggcgttgttgaaggtaatccgattatggaaatcgccaaatactttctggaatatccgctgaccattaaacgtccggaaaaatttggtggtgatctgaccgttaat
agctatgaagaactggaaagcctgtttaaaaacaaagaactgcatccgatgcgtctgaaaaatgcagttgcagaagaactgatcaaaatcctggaaccgattcgtaaacgtct
gtaa

Supplementary Note 2. Computational design

All calculations can be carried out with Rosetta3 github main repository version e6457803081372c4361f36a3ecf99e2373bbc3c5 or later.

Part 1: Command lines and input files for the matching stage

Command line:

/Users/flo/gitros/Rosetta/main/source/bin/match.macosgccrelease @1j1u_match_gen_flags -match:scaffold_active_site_residues_for_geomcsts 1j1u_3res.pos -match:geometric_constraint_file 1j1u_nd1_match_glu_no2_oh.cst -mute protocols.idealize

Input files:

1. 1j1u_match_gen_flags

```
-----------FILE BEGIN-----------------------
-database /Users/flo/gitros/Rosetta/main/database/
-s /Users/flo/pdstuff/budisa/matching/1j1u_mi.pdb
-match:lig_name ND1
-extra_res_fa /Users/flo/pdstuff/budisa/build_ligand/ensembles/rosettafied/ND1_aX.params
-use_input_sc
-match:output_format CloudPDB
-match_grouper SameSequenceAndDSPositionGrouper
-match:bump_tolerance 0.4
-output_matchres_only false
-enumerate_ligand_rotamers
-only_enumerate_non_match_redundant_ligand_rotamers
-output_virtual
----------- FILE END -----------------------
```

1j1u_mi.pdb is the Rosetta-relaxed MjTyrRS starting structure, ND1_ax.params is the p-ONB-Dopa parameters file. Both files are too big to be included in the SI, but can be provided upon request.

2. 1j1u_3res.pos

```
----------- FILE BEGIN -----------------------
N_CST 3
1: 173
2: ALL
3: ALL
----------- FILE END -----------------------
```

2. 1j1u_nd1_match_glu_no2_oh.cst

```
----------- FILE BEGIN -----------------------
# cst constraint descriptor for the constant part of the 1j1u binding site with nd1 ligand
# F.Richter



#block 1 for glutamine 173 interacting with substrate carboxylate
CST::BEGIN
  TEMPLATE::    ATOM_MAP: 1 atom_name: O3 C2 C1
  TEMPLATE::    ATOM_MAP: 1 residue3: ND1

  TEMPLATE::    ATOM_MAP: 2 atom_name: NE2 CD CG
  NATIVE
  TEMPLATE::    ATOM_MAP: 2 residue1: Q

  CONSTRAINT:: distanceAB:     2.849    0.30 180.00   0          0
  CONSTRAINT::      angle_A:   109.017   20.00 100.00   360.00   0
  CONSTRAINT::      angle_B:   122.577   20.00 100.00   360.00    0
  CONSTRAINT::   torsion_A:     52.216   10.00   50.00   360.00    0
  CONSTRAINT::   torsion_B:   144.413   15.00     0.00   360.00    0
  CONSTRAINT:: torsion_AB:   -49.393   75.00     5.00   360.00    0
CST::END

#block for an interaction with the nitro group
VARIABLE_CST::BEGIN

CST::BEGIN
  TEMPLATE::    ATOM_MAP: 1 atom_name: O4 N2 C12
  TEMPLATE::    ATOM_MAP: 1 atom_name: O5 N2 C12
  TEMPLATE::    ATOM_MAP: 1 residue3: ND1

  TEMPLATE::    ATOM_MAP: 2 atom_type: OH
  TEMPLATE::    ATOM_MAP: 2 residue1: STY

 CONSTRAINT:: distanceAB:     2.80     0.50 180.00   0
 CONSTRAINT::      angle_A:   120.00    20.00 100.00   360.00
 CONSTRAINT::      angle_B:   109.00    20.00 100.00   360.00

  ALGORITHM_INFO:: match
    SECONDARY_MATCH: DOWNSTREAM
    IGNORE_UPSTREAM_PROTON_CHI
```

```
    CHI_STRATEGY:: CHI 1 EX_THREE_THIRD_STEP_STDDEVS
    CHI_STRATEGY:: CHI 2 EX_ONE_STDDEV
  ALGORITHM_INFO::END
CST::END

CST::BEGIN

  TEMPLATE::   ATOM_MAP: 1 atom_name: O4 N2 C12
  TEMPLATE::   ATOM_MAP: 1 atom_name: O5 N2 C12
  TEMPLATE::   ATOM_MAP: 1 residue3: ND1

  TEMPLATE::   ATOM_MAP: 2 atom_type: NH2O
  TEMPLATE::   ATOM_MAP: 2 residue1: NQ

 CONSTRAINT:: distanceAB:    2.80   0.50 180.00  0
 CONSTRAINT::    angle_A:  120.00  20.00 100.00  360.00
 CONSTRAINT::    angle_B:  120.00  20.00 100.00  360.00
 CONSTRAINT::  torsion_B:  180.00  25.00   0.00  100.00   0

  ALGORITHM_INFO:: match
    SECONDARY_MATCH: DOWNSTREAM
    CHI_STRATEGY:: CHI 1 EX_THREE_THIRD_STEP_STDDEVS
    CHI_STRATEGY:: CHI 2 EX_ONE_STDDEV
    CHI_STRATEGY:: CHI 3 EX_ONE_STDDEV
  ALGORITHM_INFO::END

CST::END

CST::BEGIN
  TEMPLATE::   ATOM_MAP: 1 atom_name: O4 N2 C12
  TEMPLATE::   ATOM_MAP: 1 atom_name: O5 N2 C12
  TEMPLATE::   ATOM_MAP: 1 residue3: ND1

  TEMPLATE::   ATOM_MAP: 2 atom_name: N CA H
  TEMPLATE::   ATOM_MAP: 2 residue1: G

 CONSTRAINT:: distanceAB:    2.70   0.50 180.00  0
 CONSTRAINT::    angle_A:  120.00  40.00 100.00  360.00
 CONSTRAINT::    angle_B:  120.00  40.00 100.00  360.00
 CONSTRAINT::  torsion_B:    0.00  45.00   0.00  360.00   0

  ALGORITHM_INFO:: match
    SECONDARY_MATCH: DOWNSTREAM
  ALGORITHM_INFO::END
```

```
CST::END
VARIABLE_CST::END


#block for an interaction with the hydroxy group
VARIABLE_CST::BEGIN

CST::BEGIN
  TEMPLATE::    ATOM_MAP: 1 atom_name: O6 C7 C9
  TEMPLATE::    ATOM_MAP: 1 residue3: ND1

  TEMPLATE::    ATOM_MAP: 2 atom_type: OH
  TEMPLATE::    ATOM_MAP: 2 residue1: STY

 CONSTRAINT:: distanceAB:    2.80   0.50 180.00   0
 CONSTRAINT::     angle_A:   120.00   20.00 100.00   360.00
 CONSTRAINT::     angle_B:   109.00   20.00 100.00   360.00

  ALGORITHM_INFO:: match
    SECONDARY_MATCH: DOWNSTREAM
    IGNORE_UPSTREAM_PROTON_CHI
    CHI_STRATEGY:: CHI 1 EX_THREE_THIRD_STEP_STDDEVS
    CHI_STRATEGY:: CHI 2 EX_ONE_STDDEV
  ALGORITHM_INFO::END
CST::END

CST::BEGIN

  TEMPLATE::    ATOM_MAP: 1 atom_name: O6 C7 C9
  TEMPLATE::    ATOM_MAP: 1 residue3: ND1

  TEMPLATE::    ATOM_MAP: 2 atom_type: ONH2
  TEMPLATE::    ATOM_MAP: 2 residue1: NQ

 CONSTRAINT:: distanceAB:    2.80   0.50 180.00   0
 CONSTRAINT::     angle_A:   120.00   20.00 100.00   360.00
 CONSTRAINT::     angle_B:   120.00   20.00 100.00   360.00
 CONSTRAINT::   torsion_B:   180.00   25.00    0.00   100.00    0

  ALGORITHM_INFO:: match
    SECONDARY_MATCH: DOWNSTREAM
    CHI_STRATEGY:: CHI 1 EX_THREE_THIRD_STEP_STDDEVS
    CHI_STRATEGY:: CHI 2 EX_ONE_STDDEV
```

```
      CHI_STRATEGY:: CHI 3 EX_ONE_STDDEV
   ALGORITHM_INFO::END

CST::END

CST::BEGIN
   TEMPLATE::     ATOM_MAP: 1 atom_name: O6 C7 C9
   TEMPLATE::     ATOM_MAP: 1 residue3: ND1

   TEMPLATE::     ATOM_MAP: 2 atom_name: N CA H
   TEMPLATE::     ATOM_MAP: 2 residue1: G

  CONSTRAINT:: distanceAB:      2.70     0.50 180.00    0
  CONSTRAINT::       angle_A:   120.00    40.00 100.00   360.00
  CONSTRAINT::       angle_B:   120.00    40.00 100.00   360.00
  CONSTRAINT::   torsion_B:       0.00    45.00      0.00    360.00      0

   ALGORITHM_INFO:: match
      SECONDARY_MATCH: DOWNSTREAM
   ALGORITHM_INFO::END

CST::END

CST::BEGIN
   TEMPLATE::     ATOM_MAP: 1 atom_name: O6 C7 C9
   TEMPLATE::     ATOM_MAP: 1 residue3: ND1

   TEMPLATE::     ATOM_MAP: 2 atom_type: Ntrp
   TEMPLATE::     ATOM_MAP: 2 residue1: W

  CONSTRAINT:: distanceAB:      2.80     0.50 180.00    0
  CONSTRAINT::       angle_A:   120.00    20.00 100.00   360.00
  CONSTRAINT::       angle_B:   120.00    20.00 100.00   360.00

   ALGORITHM_INFO:: match
      SECONDARY_MATCH: DOWNSTREAM
      CHI_STRATEGY:: CHI 1 EX_TWO_HALF_STEP_STDDEVS
      CHI_STRATEGY:: CHI 2 EX_TWO_HALF_STEP_STDDEVS
   ALGORITHM_INFO::END
CST::END
VARIABLE_CST::END
----------- FILE END -----------------------
```

Part 2: Command lines and input files for the design stage

Command line:

/Users/flo/gitros/Rosetta/main/source/bin/rosetta_scripts.macosgccrelease -database /Users/flo/gitros/Rosetta/main/database/ -l no2hydroxylist1 -enzdes:cstfile 1j1u_nd1_des_glu_no2_oh.cst -parser:protocol basic_enzdes.xml @gen_flags -scorefile dessco1.txt

Input files:

1. no2hydroxylist1

- a text file containing the paths of matches to be designed

2. 1j1u_nd1_des_glu_no2_oh.cst

```
----------- FILE BEGIN -----------------------
# cst constraint descriptor for the constant part of the 1j1u binding site with nd1 ligand
# F.Richter



#block 1 for glutamine 173 interacting with substrate carboxylate
CST::BEGIN
   TEMPLATE::     ATOM_MAP: 1 atom_name: O3 C2 C1
   TEMPLATE::     ATOM_MAP: 1 residue3: ND1

   TEMPLATE::     ATOM_MAP: 2 atom_name: NE2 CD CG
   NATIVE
   TEMPLATE::     ATOM_MAP: 2 residue1: Q

   CONSTRAINT:: distanceAB:      2.849     0.30 180.00    0         0
   CONSTRAINT::       angle_A:   109.017    20.00 100.00    360.00   0
   CONSTRAINT::       angle_B:   122.577    20.00 100.00    360.00     0
   CONSTRAINT::    torsion_A:     52.216    10.00    50.00    360.00     0
   CONSTRAINT::    torsion_B:    144.413    15.00      0.00    360.00     0
   CONSTRAINT:: torsion_AB:    -49.393    75.00      5.00    360.00     0
CST::END

#block 2 for an interaction with the nitro group
VARIABLE_CST::BEGIN
```

```
CST::BEGIN
  TEMPLATE::    ATOM_MAP: 1 atom_name: O4 N2 C12
  TEMPLATE::    ATOM_MAP: 1 atom_name: O5 N2 C12
  TEMPLATE::    ATOM_MAP: 1 residue3: ND1

  TEMPLATE::    ATOM_MAP: 2 atom_type: OH
  TEMPLATE::    ATOM_MAP: 2 residue1: STY

 CONSTRAINT:: distanceAB:     2.80     0.30 180.00   0
 CONSTRAINT::      angle_A:   120.00   10.00 100.00   360.00
 CONSTRAINT::      angle_B:   109.00   10.00 100.00   360.00

  ALGORITHM_INFO:: match
    SECONDARY_MATCH: DOWNSTREAM
    IGNORE_UPSTREAM_PROTON_CHI
    CHI_STRATEGY:: CHI 1 EX_THREE_THIRD_STEP_STDDEVS
    CHI_STRATEGY:: CHI 2 EX_ONE_STDDEV
  ALGORITHM_INFO::END
CST::END

CST::BEGIN

  TEMPLATE::    ATOM_MAP: 1 atom_name: O4 N2 C12
  TEMPLATE::    ATOM_MAP: 1 atom_name: O5 N2 C12
  TEMPLATE::    ATOM_MAP: 1 residue3: ND1

  TEMPLATE::    ATOM_MAP: 2 atom_type: NH2O
  TEMPLATE::    ATOM_MAP: 2 residue1: NQ

 CONSTRAINT:: distanceAB:     2.80     0.30 180.00   0
 CONSTRAINT::      angle_A:   120.00   10.00 100.00   360.00
 CONSTRAINT::      angle_B:   120.00   10.00 100.00   360.00
 CONSTRAINT::   torsion_B:  180.00   15.00    0.00   100.00    0

  ALGORITHM_INFO:: match
    SECONDARY_MATCH: DOWNSTREAM
    CHI_STRATEGY:: CHI 1 EX_THREE_THIRD_STEP_STDDEVS
    CHI_STRATEGY:: CHI 2 EX_ONE_STDDEV
    CHI_STRATEGY:: CHI 3 EX_ONE_STDDEV
  ALGORITHM_INFO::END

CST::END
```

```
CST::BEGIN
  TEMPLATE::    ATOM_MAP: 1 atom_name: O4 N2 C12
  TEMPLATE::    ATOM_MAP: 1 atom_name: O5 N2 C12
  TEMPLATE::    ATOM_MAP: 1 residue3: ND1

  TEMPLATE::    ATOM_MAP: 2 atom_name: N CA H
  TEMPLATE::    ATOM_MAP: 2 residue1: G

 CONSTRAINT:: distanceAB:     2.70     0.50 180.00   0
 CONSTRAINT::      angle_A:   120.00   40.00 100.00   360.00
 CONSTRAINT::      angle_B:   120.00   40.00 100.00   360.00
 CONSTRAINT::   torsion_B:      0.00   45.00     0.00   360.00     0

  ALGORITHM_INFO:: match
    SECONDARY_MATCH: DOWNSTREAM
  ALGORITHM_INFO::END

CST::END
VARIABLE_CST::END


#block 3 for an interaction with the hydroxy group
VARIABLE_CST::BEGIN

CST::BEGIN
  TEMPLATE::    ATOM_MAP: 1 atom_name: O6 C7 C9
  TEMPLATE::    ATOM_MAP: 1 residue3: ND1

  TEMPLATE::    ATOM_MAP: 2 atom_type: OH
  TEMPLATE::    ATOM_MAP: 2 residue1: STY

 CONSTRAINT:: distanceAB:     2.80     0.30 180.00   0
 CONSTRAINT::      angle_A:   120.00   10.00 100.00   360.00
 CONSTRAINT::      angle_B:   109.00   10.00 100.00   360.00

  ALGORITHM_INFO:: match
    SECONDARY_MATCH: DOWNSTREAM
    IGNORE_UPSTREAM_PROTON_CHI
    CHI_STRATEGY:: CHI 1 EX_THREE_THIRD_STEP_STDDEVS
    CHI_STRATEGY:: CHI 2 EX_ONE_STDDEV
  ALGORITHM_INFO::END
CST::END

CST::BEGIN
```

```
  TEMPLATE::     ATOM_MAP: 1 atom_name: O6 C7 C9
  TEMPLATE::     ATOM_MAP: 1 residue3: ND1

  TEMPLATE::     ATOM_MAP: 2 atom_type: ONH2
  TEMPLATE::     ATOM_MAP: 2 residue1: NQ

 CONSTRAINT:: distanceAB:      2.80     0.30 180.00    0
 CONSTRAINT::       angle_A:    120.00    10.00 100.00    360.00
 CONSTRAINT::       angle_B:    120.00    10.00 100.00    360.00
 CONSTRAINT::    torsion_B:    180.00    15.00      0.00    100.00      0

  ALGORITHM_INFO:: match
    SECONDARY_MATCH: DOWNSTREAM
    CHI_STRATEGY:: CHI 1 EX_THREE_THIRD_STEP_STDDEVS
    CHI_STRATEGY:: CHI 2 EX_ONE_STDDEV
    CHI_STRATEGY:: CHI 3 EX_ONE_STDDEV
  ALGORITHM_INFO::END

CST::END

CST::BEGIN
  TEMPLATE::     ATOM_MAP: 1 atom_name: O6 C7 C9
  TEMPLATE::     ATOM_MAP: 1 residue3: ND1

  TEMPLATE::     ATOM_MAP: 2 atom_name: N CA H
  TEMPLATE::     ATOM_MAP: 2 residue1: G

 CONSTRAINT:: distanceAB:      2.70     0.50 180.00    0
 CONSTRAINT::       angle_A:    120.00    40.00 100.00    360.00
 CONSTRAINT::       angle_B:    120.00    40.00 100.00    360.00
 CONSTRAINT::    torsion_B:       0.00    45.00      0.00    360.00      0

  ALGORITHM_INFO:: match
    SECONDARY_MATCH: DOWNSTREAM
  ALGORITHM_INFO::END

CST::END

CST::BEGIN
  TEMPLATE::     ATOM_MAP: 1 atom_name: O6 C7 C9
  TEMPLATE::     ATOM_MAP: 1 residue3: ND1

  TEMPLATE::     ATOM_MAP: 2 atom_type: Ntrp
```

```
   TEMPLATE::     ATOM_MAP: 2 residue1: W

  CONSTRAINT:: distanceAB:     2.80     0.30 180.00   0
  CONSTRAINT::      angle_A:   120.00   10.00 100.00   360.00
  CONSTRAINT::      angle_B:   120.00   10.00 100.00   360.00

   ALGORITHM_INFO:: match
      SECONDARY_MATCH: DOWNSTREAM
      CHI_STRATEGY:: CHI 1 EX_TWO_HALF_STEP_STDDEVS
      CHI_STRATEGY:: CHI 2 EX_TWO_HALF_STEP_STDDEVS
   ALGORITHM_INFO::END
CST::END
VARIABLE_CST::END

#block 4 for glutamine 155 interacting with substrate amino group
CST::BEGIN
   TEMPLATE::     ATOM_MAP: 1 atom_name: N1 C1 C2
   TEMPLATE::     ATOM_MAP: 1 residue3: ND1

   TEMPLATE::     ATOM_MAP: 2 atom_name: OE1 CD CG
   TEMPLATE::     ATOM_MAP: 2 seqpos: 155
   TEMPLATE::     ATOM_MAP: 2 residue1: Q

   CONSTRAINT:: distanceAB:     2.80     0.30 180.00   0
   CONSTRAINT::      angle_A:   120.00   20.00 100.00   360.00
   CONSTRAINT::      angle_B:   120.00   20.00 100.00   360.00
CST::END


#block 5 for tyrosine 151 interacting with substrate amino group
CST::BEGIN
   TEMPLATE::     ATOM_MAP: 1 atom_name: N1 C1 C2
   TEMPLATE::     ATOM_MAP: 1 residue3: ND1

   TEMPLATE::     ATOM_MAP: 2 atom_name: OH CZ CE2
   TEMPLATE::     ATOM_MAP: 2 seqpos: 151
   TEMPLATE::     ATOM_MAP: 2 residue1: Y

   CONSTRAINT:: distanceAB:     2.90     0.30 180.00   0
   CONSTRAINT::      angle_A:   109.00   20.00 100.00   360.00
   CONSTRAINT::      angle_B:   109.00   20.00 100.00   360.00
CST::END
```

```
#block 6 for glutamine 173 interacting with substrate amino group
CST::BEGIN
   TEMPLATE::     ATOM_MAP: 1 atom_name: N1 C1 C2
   TEMPLATE::     ATOM_MAP: 1 residue3: ND1

   TEMPLATE::     ATOM_MAP: 2 atom_name: OE1 CD CG
   TEMPLATE::     ATOM_MAP: 2 identical: 1 2
   TEMPLATE::     ATOM_MAP: 2 residue1: Q

   CONSTRAINT:: distanceAB:      2.80     0.30 180.00    0
   CONSTRAINT::       angle_A:   109.00    20.00 100.00    360.00
   CONSTRAINT::       angle_B:   120.00    20.00 100.00    360.00
CST::END

#block 7 for glutamine 173 - glutamine 155 interaction
CST::BEGIN
   TEMPLATE::     ATOM_MAP: 1 atom_name: OE1 CD CG
   TEMPLATE::     ATOM_MAP: 1 identical: 1 2
   TEMPLATE::     ATOM_MAP: 1 residue1: Q

   TEMPLATE::     ATOM_MAP: 2 atom_name: NE2 CD CG
   TEMPLATE::     ATOM_MAP: 2 identical: 4 2
   TEMPLATE::     ATOM_MAP: 2 residue1: Q

   CONSTRAINT:: distanceAB:      2.80     0.30 180.00    0
   CONSTRAINT::       angle_A:   125.00    20.00 100.00    360.00
   CONSTRAINT::       angle_B:   120.00    20.00 100.00    360.00
CST::END
----------- FILE END -----------------------
```

3. basic_enzdes.xml

```
----------- FILE BEGIN -----------------------
<dock_design> //Only things within "less-than" and "greater-than" signs are read
        <SCOREFXNS>
                <enzdes weights=enzdes/> //name is enzdes, weights=X where X is a .wts file from database
                <talaris weights=talaris2013 >
                        <Reweight scoretype=coordinate_constraint weight=1.0 />
                      <Reweight scoretype=atom_pair_constraint weight=1.0 />
                      <Reweight scoretype=angle_constraint weight=1.0 />
                      <Reweight scoretype=dihedral_constraint weight=1.0 />
                </talaris>
              <softpack weights=ligand_soft_rep >
```

```
                <Reweight scoretype=coordinate_constraint weight=1.0 />
                <Reweight scoretype=atom_pair_constraint weight=1.0 />
                <Reweight scoretype=angle_constraint weight=1.0 />
                <Reweight scoretype=dihedral_constraint weight=1.0 />
                <Reweight scoretype=hbond_sc weight=1.3 />
                <Reweight scoretype=hbond_bb_sc weight=1.3 />
            </softpack>
        </SCOREFXNS>
        <TASKOPERATIONS>
            <DetectProteinLigandInterface name="interf_resfile" cut1=7.0 cut2=9.0 cut3=11 cut4=13
resfile="../1j1u_const.resfile" />
            <DetectProteinLigandInterface name="interf_repack" cut1=0.0 cut2=0.0 cut3=12 cut4=14 repack_only=1
design=0 />
            <AddRigidBodyLigandConfs name="ligandrb" />
            <SetCatalyticResPackBehavior name="catrespack" />
            <LimitAromaChi2 name="limaro2" />
            <InitializeFromCommandline name="cmdline" />
        </TASKOPERATIONS>
        <FILTERS> //define filters here; order of definition is NOT important
            EnzdesScorefileFilter name="enz_sco1" requirements="ech13loopreqs1.txt" />
            EnzdesScorefileFilter name="enz_sco2" requirements="ech13loopreqs2.txt" />
            <ResidueConformerFilter name="ligconf" restype="ND1" />
        </FILTERS>

    <MOVERS>//define movers; order of definition is NOT important
            <AddOrRemoveMatchCsts name=cstaddnew cst_instruction="add_new" accept_blocks_missing_header=1
/>
            <AddOrRemoveMatchCsts name=cstadd cst_instruction="add_pregenerated"/>
            <AddOrRemoveMatchCsts name=cstremove cst_instruction="remove" keep_covalent=1/>
            <EnzRepackMinimize name=cst_opt design=1 scorefxn_minimize=talaris scorefxn_repack=talaris
minimize_rb=1 minimize_sc=1 minimize_bb=1 cycles=1 cst_opt=1 task_operations=interf_resfile,ligandrb,catrespack,limaro2
/>
            <EnzRepackMinimize name=desmin design=1 scorefxn_minimize=talaris scorefxn_repack=talaris
minimize_rb=1 minimize_sc=1 minimize_bb=1 cycles=3 task_operations=interf_resfile,ligandrb,catrespack,limaro2 />
           EnzRepackMinimize name=softdesmin design=1 scorefxn_minimize=enzdes scorefxn_repack=softpack
minimize_rb=1 minimize_sc=1 minimize_bb=1 cycles=1 task_operations=interf_resfile,ligandrb,catrespack,limaro2 />
            <EnzRepackMinimize name=repackmin repack_only=1 scorefxn_minimize=talaris scorefxn_repack=talaris
minimize_rb=1 minimize_sc=1 minimize_bb=1 cycles=1 task_operations=interf_repack,ligandrb,limaro2 />
            <ScoreMover name=score scorefxn=talaris verbose=0 />
    </MOVERS>
    <APPLY_TO_POSE> //could also add PSSM csts here
    </APPLY_TO_POSE>
    <PROTOCOLS>// construct your own protocol; order of definition IS important. It determines workflow
           <Add mover_name=cstaddnew/>
```

```
            <Add mover_name=cst_opt />
           <Add mover_name=desmin />
           <Add mover_name=cstremove/>
            <Add mover_name=repackmin/>
            <Add mover_name=cstadd/>
            <Add mover_name=score />
            <Add filter_name=ligconf />
      </PROTOCOLS>
</dock_design>
----------- FILE END -----------------------
```

3. gen_flags

```
----------- FILE BEGIN-----------------------
-database /Users/flo/gitros/Rosetta/main/database
-score:weights talaris2013_cst
-extra_res_fa /Users/flo/pdstuff/budisa/build_ligand/ensembles/rosettafied/ND1_aX.params
-nstruct 25
-ex1
-ex2
-ex_catalytic_rot 5
-final_repack_without_ligand
-packing:linmem_ig 10
#-packing:multi_cool_annealer 10
-run:nblist_autoupdate true
-use_input_sc
#note: the following 4 flags are only necessary for repacking w/o ligand to work, the taskops in the xml are not sensitive
-cut1 0.0
-cut2 0.0
-cut3 16.0
-cut4 20.0
-detect_design_interface
-favor_native_res 0.5
-MM:ignore_missing_bondangle_params
-mute protocols.BumpGrid protocols.forge.components.VarLengthBuild protocols.moves.MonteCarlo
protocols.packstat.SimplePDB protocols.match.Matcher protocols.toolbox.RotamerSetOperations.RigidBodyMoveRotSetOps
core.scoring.NeighborList core.pack.dunbrack.SingleLigandRotamerLibrary
protocols.match.downstream.LigandConformerBuilder core.mm.MMBondAngleLibrary protocols.idealize
core.fragment.picking_old core.conformation.Residue protocols.toolbox.match_enzdes_util
protocols.forge.remodel.RemodelLoopMover protocols.forge.remodel.RemodelLoopMover
protocols.forge.build.BuildManager protocols.loops.loops_main core.pack.task: Packer task
core.pack.interaction_graph.interaction_graph_factory core.pack.pack_rotamers
protocols.match.upstream.ProteinUpstreamBuilder protocols.match.downstream.SecondaryMatcherToDownstreamResidue
protocols.toolbox.match_enzdes_util
```

```
-run:preserve_header
-enzdes:parser_read_cloud_pdb
-jd2:enzdes_out
----------- FILE END -----------------------
```

Part 3: Filter criteria file

```
----------- FILE BEGIN -----------------------
output sortmin ligconf
#output sortmin SR_9_interf_E_1_2
req total_score value < -321.0
req tot_burunsat_pm value < 88
req tot_NLconts_pm value > 49
req all_cst value < 10.0
req SR_9_interf_E_1_2 value < -11.0
req nlr_totrms value < 1.0
req SR_1_all_cst value < 2.0
req nlr_SR1_rms value < 1.0
req nlr_SR2_rms value < 1.0
req nlr_SR3_rms value < 1.0
req nlr_SR4_rms value < 1.0
req nlr_SR5_rms value < 1.0
----------- FILE END -----------------------
```

Raw Rosetta3 enzdes sequence profile for 49 structures selected for aaRS gene library generation:

Y32:	0.84 A,	0.10 S,	0.06 G								
I33:	0.08 A,	0.04 I,	0.02 N,	0.16 S,	0.29 T,	0.41 V					
I63:	0.59 I,	0.02 M,	0.39 V								
I64:	0.06 I,	0.94 V									
L65:	0.20 A,	0.08 F,	0.37 I,	0.02 H,	0.04 M,	0.06 L,	0.02 S,	0.02 T,	0.02 W,	0.12 V,	0.04 Y
L66:	0.06 I,	0.94 L									
A67:	0.78 A,	0.14 Q,	0.02 M,	0.06 G							
L69:	0.02 A,	0.08 G,	0.02 H,	0.04 K,	0.20 L,	0.16 Q,	0.02 S,	0.02 T,	0.43 W		
H70:	0.22 A,	0.04 E,	0.02 G,	0.65 N,	0.02 Q,	0.04 S					
V103:	0.20 I,	0.08 K,	0.12 L,	0.12 R,	0.04 T,	0.43 V					
G105:	0.27 Q,	0.18 A,	0.04 S,	0.51 G							
F108:	0.08 I,	0.02 Y,	0.04 T,	0.08 L,	0.78 F						
Q109:	0.84 Q,	0.10 A,	0.04 Y,	0.02 E							
M154:	0.04 A,	0.18 E,	0.02 D,	0.04 G,	0.10 K,	0.06 M,	0.04 N,	0.02 Q,	0.49 T		
V156:	0.22 A,	0.29 I,	0.49 V								
N157:	0.49 A,	0.22 G,	0.29 N								
D158:	0.49 A,	0.08 S,	0.43 G								
I159:	0.41 A,	0.18 G,	0.14 I,	0.12 L,	0.06 Q,	0.06 S,	0.02 T				
L162:	0.14 A,	0.16 E,	0.02 F,	0.43 M,	0.06 L,	0.06 Q,	0.02 S,	0.02 T,	0.08 V		
V164:	0.35 A,	0.06 D,	0.02 Q,	0.02 P,	0.08 S,	0.14 T,	0.33 V				
A167:	0.06 A,	0.31 G,	0.08 N,	0.10 Q,	0.41 S,	0.04 T					
I176:	0.51 I,	0.24 A,	0.02 T,	0.22 V							
H177:	0.10 A,	0.12 D,	0.10 G,	0.02 L,	0.24 N,	0.29 Q,	0.04 S,	0.08 Y			
A180:	0.78 A,	0.20 Q,	0.02 G								
V188:	0.16 A,	0.02 D,	0.24 H,	0.08 M,	0.02 L,	0.02 N,	0.20 Q,	0.02 P,	0.02 S,	0.14 T,	0.06 V
C190:	0.22 A,	0.04 Q,	0.67 C,	0.04 L,	0.02 N						

Library size: 3*6*3*2*11*2*4*9*6*6*4*5*4*9*3*3*3*7*9*7*6*4*8*3*11*5 ≈ $8.4 * 10^{17}$

Supplementary References

1. Nguyen, D.P.; Mahesh, M.; Elsässer, S.J.; Hancock, S.M.; Uttamapinant, C.; Chin, J.W. Genetic encoding of photocaged cysteine allows photoactivation of TEV protease in live mammalian cells. *J. Am. Chem. Soc.* **2014**, *136*, 2240–3.
2. Böcker, J.K.; Dörner, W.; Mootz, H.D. Light-control of the ultra-fast Gp41-1 split intein with preserved stability of a genetically encoded photo-caged amino acid in bacterial cells. *Chem. Commun.* **2019**, *55*, 1287–1290.
3. Hauf, M.; Richter, F.; Schneider, T.; Faidt, T.; Martins, B.M.; Baumann, T.; Durkin, P.; Dobbek, H.; Jacobs, K.; Möglich, A.; et al. Photoactivatable Mussel-Based Underwater Adhesive Proteins by an Expanded Genetic Code. *Chembiochem* **2017**, *18*, 1819–1823.
